# BRB-ArrayTools Data Archive for Human Cancer Gene Expression: A Unique and Efficient Data Sharing Resource

**DOI:** 10.4137/cin.s448

**Published:** 2008-04-21

**Authors:** Yingdong Zhao, Richard Simon

**Affiliations:** Biometric Research Branch, National Cancer Institute, National Institutes of Health, Rockville, Maryland, U.S.A

## Abstract

The explosion of available microarray data on human cancer increases the urgency for developing methods for effectively sharing this data among clinical cancer investigators. Lack of a smooth interface between the databases and statistical analysis tools limits the potential benefits of sharing the publicly available microarray data. To facilitate the efficient sharing and use of publicly available microarray data among cancer investigators, we have built a BRB-ArrayTools Data Archive including over one hundred human cancer microarray projects for 28 cancer types. Expression array data and clinical descriptors have been imported into BRB-ArrayTools and are stored as BRB-ArrayTools project folders on the archive. The data archive can be accessed from: http://linus.nci.nih.gov/~brb/DataArchive.html Our BRB-ArrayTools data archive and GEO importer represent ongoing efforts to provide effective tools for efficiently sharing and utilizing human cancer microarray data.

## Background

Since its first appearance in the late 1990’s, microarray technology has been applied in almost all areas of biology and medicine. Gene expression experiments have been proven to be very powerful in cancer diagnosis and treatment [1,2]. The explosion of available microarray data on human cancer increases the urgency for developing methods for effectively sharing this data among cancer clinical investigators.

As we know, data sharing not only allows the research community to validate original analyses, but also to enable new analyses, design new methodology and perform meta-analysis on data generated from different laboratories [3,4]. There are two major components to data sharing effectiveness. One is ease of access (deposit and download) to the publicly available data. This objective is closely related to the effectiveness of the database as an archive. The second component is how easily and effectively the archived data can be analyzed by the community.

Unlike sequence data which is successfully shared by research community via public sequence databases and online search tools, DNA microarray expression data are much more complex. Multiple steps are involved in the data generation, many experimental platforms are widely used and data can be deposited with different levels of processing (for example, log intensity, log ratio, intensity, or probe set level vs. probe level). Microarray data analysis is also statistically complex. Currently, efforts to build publicly available gene expression databases have been successfully as archives only. For example, NCBI’s Gene Expression Omnibus (GEO), EBI’s ArrayExpress and the Stanford Microarray Database (SMD) support MIAME compliant data submissions and provide online resource for gene expression data browsing, query and retrieval. However, they provide only limited statistical analysis capabilities. Sophisticated statistical analysis software for microarray data analysis are available as stand-alone tools. Users often find it difficult to load the data into external analysis tools because of in-compatibility of the data format. Although more and more stand alone statistical packages have been designed specifically for gene expression data analysis, the user interfaces are not well designed to accommodate publicly available microarray data. Lack of a smooth interface between the databases and statistical analysis tools limits the potential benefits of sharing the publicly available microarray data.

BRB-ArrayTools is a comprehensive state-of-the-art statistical analysis system for the analysis of microarray gene expression data [5]. The ArrayTools package is portable and not tied to any database. BRB-ArrayTools contains numerous analysis tools, including SAM, multivariate permutation tests, classification analysis with complete cross-validation, survival prediction analysis and gene set enhancement analysis. It incorporates state-of-the-art statistical methods for analysis of microarray data but the methods are menu driven and the user interface is Excel based and accessible to biomedical scientists. BRB-ArrayTools is computationally very efficient, using compiled modules running transparently to the user. It can handle projects with up to 1000 arrays and 100,000 probe sets per array. It now has over 7000 registered users from about 2000 institutions in 68 countries around the world. Researchers have been using BRB-ArrayTools to do microarray analysis and published over 500 peer-reviewed papers. BRB-ArrayTools has been proven to be a very powerful tool for microarray analysis. To facilitate the efficient sharing and use of publicly available microarray data among cancer investigators, we have built a BRB-ArrayTools Data Archive for Human Cancer Human Gene Expression.

## Construction and Content

### Data archive content and infrastructure

The data sets included are all from publicly available microarray data resources such as GEO, ArrayExpress and SMD [6–9]. We were not able to collect any datasets from the Oncomine site as it does not support download of raw data [10]. Our data archive currently includes 109 human cancer microarray projects for 28 cancer types, which include more than 6000 microarrays. Each project consists as least 10 samples, along with useful clinical and pathological phenotype information. The citations and the PDF files for the publication of each data set are also listed and linked to PUBMED. There is a README file attached to each record explaining the study purpose and data information. Expression array data and clinical descriptors have been imported into BRB-Array-Tools and are stored as BRB-ArrayTools project folders on the archive.

### Data access

To analyze a dataset, simply download and unzip the file from http://linus.nci.nih.gov/~brb/DataArchive.html

The unzipped archive contains a project worksheet which the user opens in Excel on a Microsoft Windows PC on which BRB-ArrayTools has been installed. To download BRB-ArrayTools, go to http://linus.nci.nih.gov/~brb/DataArchive.html

### Data deposition and management

We update the data archive frequently to include new publicly available datasets from NCBI GEO, EBI ArrayExpress and Stanford MicroArray Database. We also encourage BRB-ArrayTools users to deposit their datasets directly. To deposit a dataset, users just need to create a README file, using the sample README file as a template and upload it with their BRB-ArrayTools project folder to brb@linus.nci.nih.gov.

For inclusion in the Data Archive we require that the dataset be based on profiling human cancer tissue samples, contain more than 10 samples, and contain clinical phenotype information. Some degree of quality assessment on the data is provided in the process of creating BRB-ArrayTools projects from archived or submitted datasets. Enabling users to subject these datasets to their independent analyses provides some quality checks on the initial publications.

### Example

We chose one lung cancer data set (Beers et al. [11]) as an example to illustrate use of the archive with BRB-ArrayTools to do comprehensive statistical analysis.

**Figure 1:**
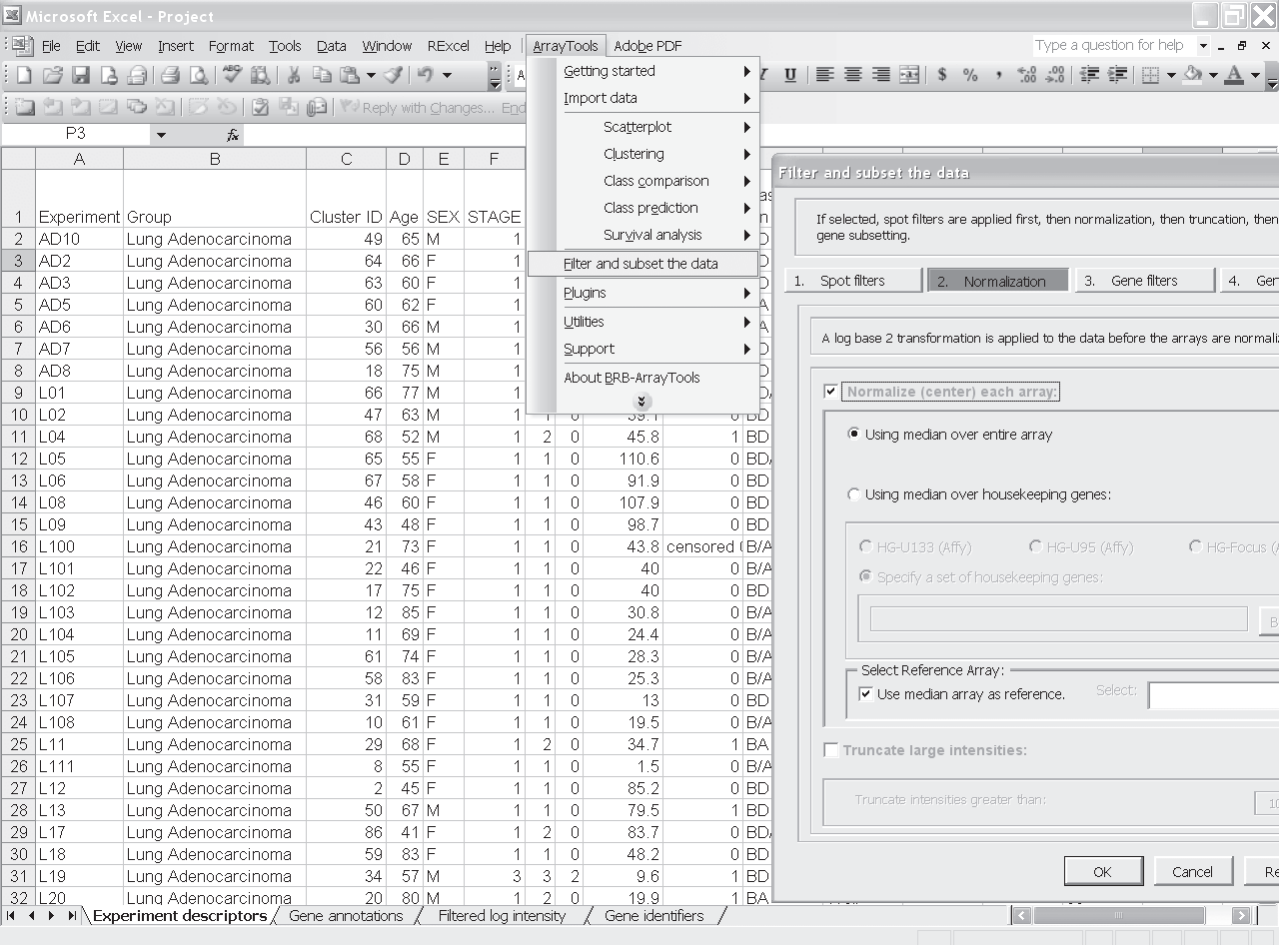
Screen shot of experiment descriptor sheet and data normalization menu (right).

**Figure 2:**
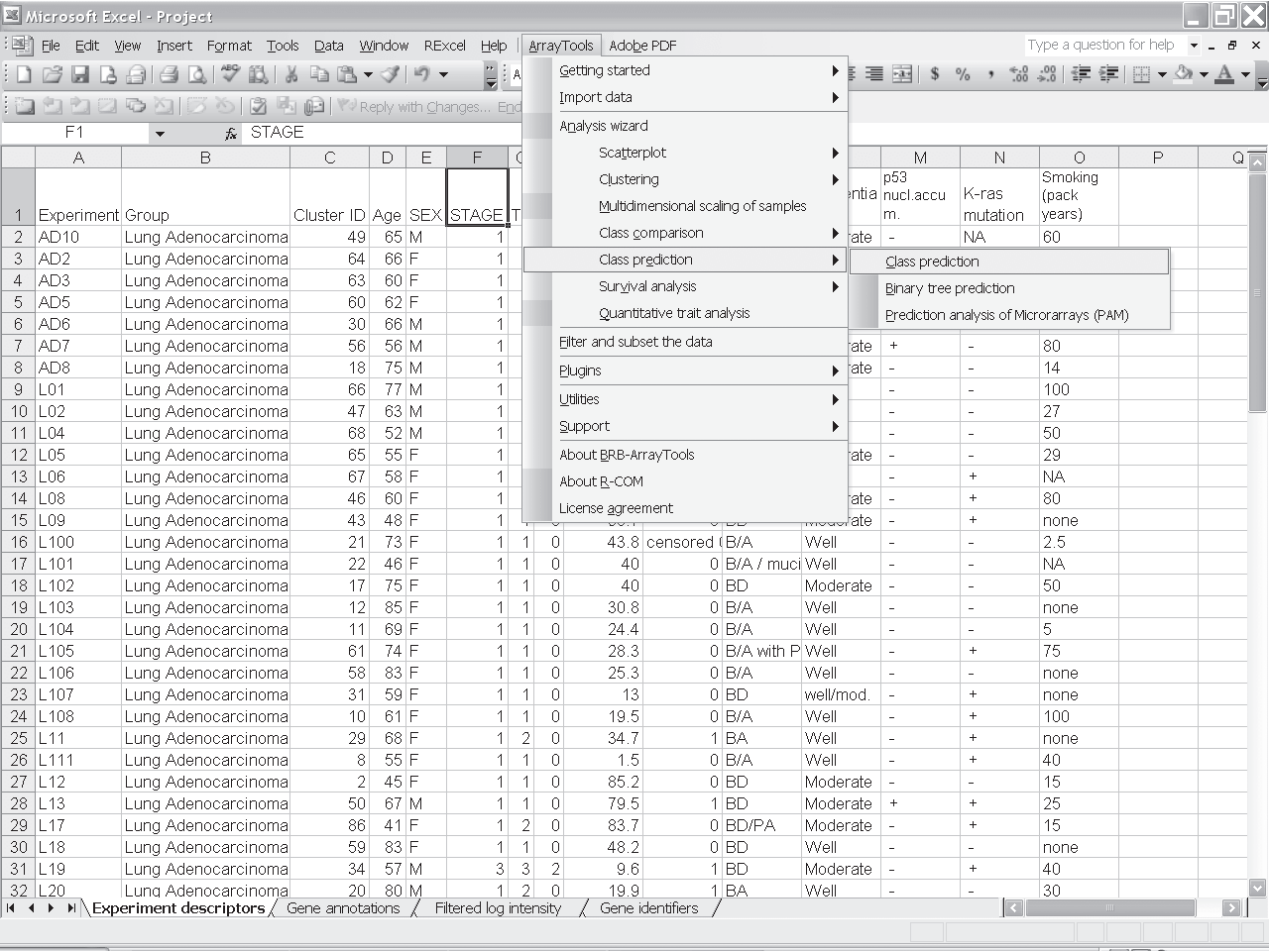
Screen shot of select class prediction in BRB-ArrayTools menu.

**Figure 3:**
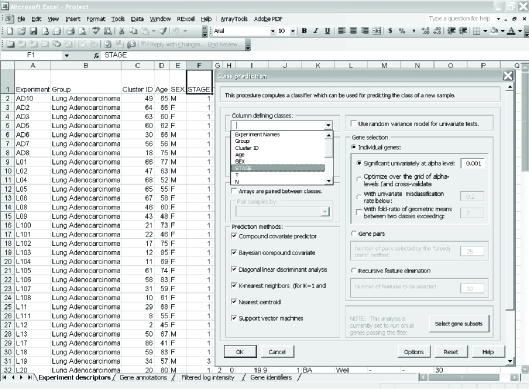
Screen shot of the parameter settings dialog in class prediction function menu.

**Figure 4:**
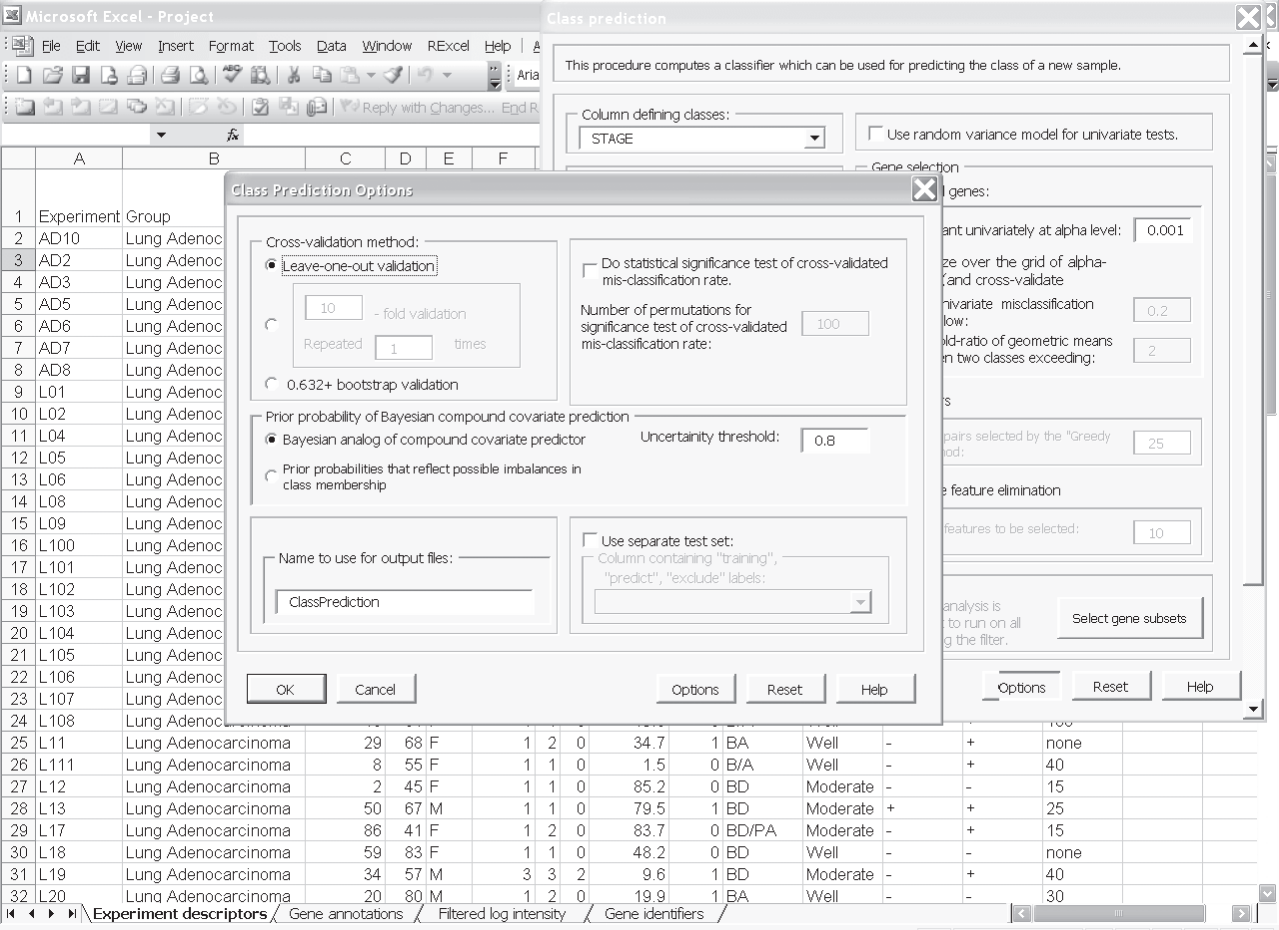
Screen shot of the options page of the class prediction set-up dialog.

**Figure 5:**
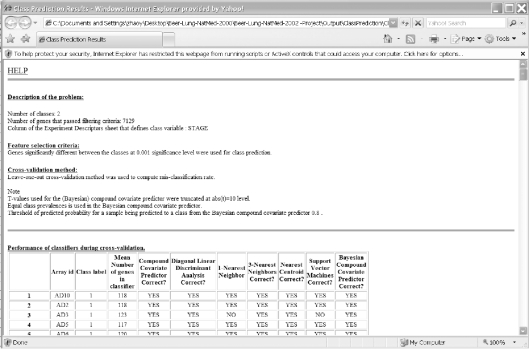

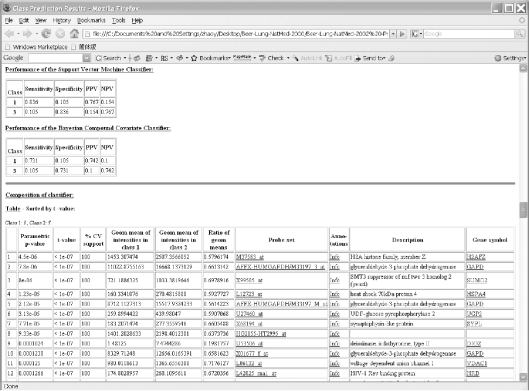
Figure 5A and 5B. Screen shot of the output html file for class prediction results.

1. Click on the zip file link in the data archive web page and save the dataset on your computer. Extract the zip file into a new folder and click on the file named “Project.xls” to open it in Excel. The project file contains four sheets: experiment descriptors, gene annotations, filtered log intensity and gene identifiers. The experiment descriptors sheet contains clinical information associated with each of the arrays. Each row of the experiment descriptor sheet corresponds to an array and there is a column for each type of clinical descriptor. The descriptors are different for different projects, depending on what information was made available by the authors. For the Beers dataset, the experiment descriptors sheet is shown in Figure 1 and contains age, sex, stage, smoking history, survival time, survival censoring indicator, etc. The filtered log intensity worksheet for the current release of BRB-ArrayTools no longer shows the expression data because that data is saved in binary files in the project folder in order to avoid limitations of Excel on numbers of rows and columns. Any desired subset of the expression data can be displayed in the filtered log intensity sheet by clicking on “Click to display the data” on the top left of the sheet.
2. Data normalization needs to be done before analysis. We have created the projects with raw unnormalized data so that users can normalize as they wish. BRB-ArrayTools provides several normalization options. They are available under the “filter and subset the data” submenu of the BRB-ArrayTools menu on the Excel menu bar. The menu options available for dual label arrays are somewhat different than for single label arrays like Affymetrix GeneChips™ arrays but in creating the projects we have specified to BRB-ArrayTools what array platform was used. The Beers dataset was based on Affymetrix arrays and for this example we have selected “using median over entire array” and “use median array as reference” for normalization (Fig. 1 right side).
3. Suppose the user wants to build a classifier for predicting tumor stage based on gene expression profile. Clicking on the class prediction entry under the BRB-ArrayTools menu (Fig. 2) brings up the class prediction set-up dialog (Fig. 3). “STAGE” is chosen as “Column defining class” using the pull down menu. The pull-down menu will show labels for all columns in the experiment descriptors sheet. There are many options possible for class prediction but the default settings have been carefully selected to be useful for many applications. By default the program will develop predictive classifiers using six different methods including the compound covariate method of Radmacher et al. [12], diagonal linear discriminant analysis, nearest neighbor and nearest centroid classification and support vector machines. One part of developing a predictive classifier is selecting the genes to be included and BRB-ArrayTools provides several options for that also. The default is to select genes differentially expressed among classes at a significance level 0.001. More complex methods like recursive feature elimination are also available. The options page of the class prediction set-up dialog is shown in Figure 4. There are several options for internal validation of the predictive classifier. The default is complete leave-one-out cross-validation. “Complete” means that for each loop of the cross-validation the informative genes are selected for the restricted training set not containing the omitted case and the six classifiers re-fitted using those genes. If the user wants to repeat the complete cross-validation with the class labels permuted to determine whether the cross-validated prediction accuracy is better than chance, the box at the top right of the option dialog (Fig. 4) would be checked. Here all of the options can be left at their default values; the user needs only to have given the column defining the classes of interest and possibly the name to be used for the output file that will save the results of the analysis.
4. The class prediction analysis, including complete cross-validation, will usually be complete within a few minutes or less and results will be shown by the program automatically opening an html file containing the output (Fig. 5A and 5B). The first table shows performance of the classifiers during cross-validation for each of the prediction methods. Subsequent tables show cross-validated sensitivity, specificity, positive and negative predictive values for the classifiers and information about the genes included in the classifiers. The genes shown are those based on fitting the classifier to the full dataset, but the column labeled “% CV support” shows what proportion of the cross-validation loops contained each gene in the classifiers. The genes included in the classifier for the full dataset are sorted by t-value with a link to view the actual expression of significant genes. Parameters for the prediction rules for the linear classifiers are also shown on the html file. The details of the output file for this example can be seen at http://linus.nci.nih.gov/Data/zhaoy/cancerinfo/example/ClassPrediction.html

## Discussion

We have also built a Gene Expression Omnibus (GEO) import tool for BRB-ArrayTools. GEO currently represents the largest single resource for publicly available gene expression data. This importer allows the users to automatically import the GDS dataset file in the NCBI Gene Expression Omnibus database into BRB-ArrayTools. A GDS dataset is a text file in SOFT format that represents a curated collection of biologically and statistically comparable GEO Samples reassembled by NCBI staff. Currently there are more than 1500 GDS data sets in GEO database. To import the GEO dataset file into BRB-ArrayTools, the users only need to input the GDS number which can be retrieved from NCBI GEO website. The GEO importer therefore serves us as an efficient pipeline to collect the most recent publicly available data in the GEO database.

## Conclusions

In conclusion, our BRB-ArrayTools data archive and GEO importer represent ongoing efforts to provide effective tools for efficiently sharing and utilizing human cancer microarray data. By combining BRB-ArrayTools with the Data Archive, cancer investigators can re-analyze or extend the analysis of publicly available data or combining the publicly available data with their own data set to do external validation analysis. The fact that the Data Archive includes clinical annotations of specimens in an “experiment descriptor file” ready for use in BRB-ArrayTools makes the resource especially valuable.
